# Impact of zero-markup consumable policy and national procurement of coronary stents on hospitalization expenses: an interrupted time series analysis

**DOI:** 10.3389/fpubh.2025.1364116

**Published:** 2025-01-30

**Authors:** Shao-long Wu, Kun Wang, Xin Yang, Ning Liu, Wen-xin Zou, Xiao-wei Li, Dan-hua Huang, Tian-hao Yu

**Affiliations:** ^1^Department of Public Administration, School of Government, Sun Yat-sen University, Guangzhou, China; ^2^Sun Yat-sen Global Health Institute, Institute of State Governance, Sun Yat-sen University, Guangzhou, China; ^3^Department of Medical Statistics, School of Public Health, Sun Yat-sen University, Guangzhou, China; ^4^Department of Health Policy and Management, School of Public Health, Sun Yat-sen University, Guangzhou, China; ^5^School of Management, Lanzhou University, Lanzhou, China; ^6^China Research Center for Government Performance Management, Lanzhou University, Lanzhou, China; ^7^Department of Cardiology, The Affiliated Guangdong Second Provincial General Hospital of Jinan University, Guangzhou, China; ^8^School of Public Health, Guangzhou Medical University, Guangzhou, China

**Keywords:** consumable, coronary stents, hospitalization expenses, zero-markup policy, national procurement, China

## Abstract

**Background:**

After the successful reforms of drug pricing, China has implemented two significant reforms to reduce medical consumables expenses: the elimination of markup on prices (zero-markup policy) and the implementation of centralized procurement of high-value medical consumables (national procurement policy). The public hospitals in Guangdong province had implemented the zero-markup policy and national procurement policy for coronary stents. Currently, research on the zero-markup consumable policy and national procurement of coronary stents for medical consumables at the hospital level lacks analysis of changes in cost structures (other expenses) and studies on hospital response strategies.

**Objective:**

This study aims to examine the effects of these reforms on hospitalization expenses for patients undergoing coronary stent implantation, including the specific changes in total hospitalization expenses, the component of total expenses, and the proportion of each expense category following the implementation of the reforms.

**Methods:**

Data were obtained from the case management system of Guangdong Provincial Second People’s Hospital in China. An interrupted time series design was employed to assess the impact of the reforms on inpatient expenses.

**Results:**

Before the reforms, the median total hospitalization expense was 63,124.98 CNY, which decreased to 58,332.50 CNY during the zero-markup consumable policy period and further decreased to 48,266.34 CNY during the national centralized procurement policy period. The median expenses for western drugs, diagnostic tests, treatment, surgery, medical consumables, and other categories also displayed varying patterns. The expenses for medical consumables notably decreased from 39,119.52 CNY to 785.79 CNY after the implementation of the national procurement policy. The slope change of medical consumables expenses decreased by 600.81 CNY per month after the zero-markup policy, and the immediate effect of the national procurement policy resulted in a decrease of 32,160.81 CNY in median medical consumables expenses. Other expense categories experienced notable changes, with a substantial increase in the median of other expenses by 28,350.39 CNY during the national procurement policy period.

**Conclusion:**

The reforms in medical consumables pricing and procurement have effectively reduced the cost of consumables for inpatients undergoing coronary stent plantation. The analysis of interrupted time series revealed that the elimination of the consumable markup policy had a limited effect on the level and trend of total hospitalization expenses. However, the implementation of the national procurement policy for coronary stents led to a significant reduction in medical consumables expenses. The study found that after the reform, hospitals mitigated revenue losses by increasing ambiguous other expenses, which may involve the shifting of medical consumable expenses in their accounting records.

## 1 Introduction

In China, the medical services are delivered mainly by public hospitals (1). In the 1980s, when China transformed its centrally planned economy into a market-oriented system, the government reduced its investment in public hospitals (2). The financial subsidies to public hospitals decreased from around 40% of the total expenditure before economic reform to around 10% in the early 1990s (3, 4). However, to ensure the affordability of medical services, the government continued to strictly control the costs of surgery, nursing, examinations, laboratory tests, and registration in public hospitals and introduced inappropriate incentives to compensate for the reduction in government investment (3).

These incentives allowed public hospitals to generate revenue through fee-for-service method and mainly profit from a 15% makeup on the cost of drugs and a 10% makeup on the cost of consumables (1, 3). Meanwhile, the incentives allowed doctors to receive additional bonuses based on the income they generated. This distorted compensation system and imperfect payment system are believed to be the cause of excessive growth in healthcare expenses (5). In addition to insufficient medical insurance coverage and low reimbursement rates, the public often complains about the high cost of accessing healthcare services (6).

To tackle the problem of expensive healthcare costs, the Chinese government launched a new round of healthcare reform in 2009. As the main providers of healthcare services in China, the primary objective of the public hospital reform was to enhance their character of public welfare (7, 8). The reform entailed the elimination of inappropriate incentives such as the mark-up policy on drugs and consumables, as well as the increase in medical service expenses. The central government has taken a series of measures to curb the increase in drug costs. By September 30, 2017, the Chinese government had abolished the drug markup policy for all public hospitals.

The zero-markup drug policy required public hospitals to control the proportion of drug revenue to around 30% (9, 10). In 2012, the proportion of drug revenue in public hospitals was around 40% of the total revenue. Since the drug markup policy, the proportion of drug revenue in public hospitals has gradually declined to around 27% of total revenue in 2019 (11, 12). The loss of public hospital revenue was covered by service price increases paid by the medical insurance fund, local government financial subsidy, and the hospital’s own revenue generation, accounting for 80, 10, and 10%, respectively. After the implementation of the zero-markup policy for drugs, the central government implemented a national centralized drug procurement program to further reduce drug prices and improve the safety of drugs (13).

However, the problem of public hospitals relying on revenue from drugs and consumables has not been thoroughly resolved, and the profit-oriented behavior of public hospitals has not been thoroughly reversed. In response to a series of drug price reforms, public hospitals have been trying various approaches to offset the revenue loss. Public hospitals may shift profit from drugs to high-value medical consumables and medical examinations (14).

Liu et al. (14) found in their study in Beijing that public hospitals may increase the volume of CT and MRI services to make up for the loss, while Fu et al. (15) and Gao et al. (16) reported in their studies in Beijing that consumable expenses increased after the implementation of the zero-markup drug policy. The proportion of drug revenue in public hospitals decreased from around 40% of total revenue in 2012 to around 27% in 2019, while the proportion of consumables revenue increased from around 13% in 2012 to around 21% in 2019 (11, 12). For instance, patients who received coronary intervention treatment consume a lot of surgical consumables and have a heavy economic burden.

To ensure the success of abolishing the drug price markup policy and to curb the shift of patient expense burden from drugs to medical consumables, it is imperative to abolish the price markup policy on medical consumables. Currently, there is a wealth of research on the cancelation of markup policies and centralized procurement policies on drugs, but there is little research addressing the cancellation of markup policies and centralized procurement policies on medical consumables. We analyze the impact of these two policies on patients and hospitals using data from a single hospital.

## 2 Background

According to the China Health Statistical Yearbook 2021 (17), the mortality rate of coronary heart disease in China in 2020 was 126.91/100,000 for urban residents and 135.88/100,000 for rural residents, and the mortality rate of coronary heart disease in 2020 continued the upward trend since 2012. In 2019, the total hospitalization expense for ischemic heart disease is 125.625 billion yuan, including 42.784 billion yuan for angina pectoris and 32.118 billion yuan for myocardial infarction.

According to the first page data of 10,259,521 cardiovascular disease-related inpatient cases from 1,910 tertiary public hospitals (79.5% of the number of tertiary public hospitals nationwide) and 2,124 secondary public hospitals (35.9% of the number of secondary public hospitals nationwide) that conducted cardiovascular disease diagnosis and treatment (excluding military and traditional Chinese medicine hospitals), a total of 1,014,266 patients in China received percutaneous coronary intervention (PCI) treatment in 2020 (18). 597,460 patients admitted for acute myocardial infarction had a per capita hospitalization expense of 30,171 yuan, of which the per capita treatment expense was 2,475.7 yuan (8.21%), the per capita surgery expense was 4,466.6 yuan (14.80%), and the per capita medical consumables expense was 13,990.4 yuan (46.37%) (17). The cost structure is unreasonable because the sum of treatment and surgery costs was less than the medical consumables cost, which accounts for almost half of the hospitalization cost.

In a study conducted in the United States, from 2010 to 2013, 11,969 myocardial infarction patients who underwent percutaneous coronary intervention treatment in 233 hospitals had an average hospitalization cost of $19,327 for ST-elevation myocardial infarction patients and an average hospitalization cost of $18,465 for non-ST-elevation myocardial infarction patients. Of hospitalization costs, 45% were related to catheterization laboratory expenses (including device implantation), 22% were related to room and board, 14% were related to supplies, and 9% were related to pharmacy expenses (19).

In China, the coronary artery stenting procedure requires the collaboration of four medical personnel wearing lead aprons weighing approximately 15 pounds in an X-ray environment. Compared to a few years ago when doctors would receive only a few hundred yuan, they can now earn a compensation of 1,500 yuan for performing the surgery (20). Compared with the highest cost of interventional surgery in the United States, both the amount and proportion of surgical service expenses in China are too low. However, public hospitals and doctors, especially departments that use a large number of consumables, can generate revenue by using more consumables due to the existence of the markup policy on consumables. Due to this imperfect compensation mechanism, doctors cannot earn income through their skills and services. Hospitals need to ensure sufficient operating costs to survive, and have to rely on high-value consumables and other methods to make up for this part of the loss. As a result of this flawed policy, the national health insurance department and patients also bear a heavy financial burden. Both doctors and patients are not satisfied as they should be.

In July 2019, the State Council introduced a policy for high-value medical consumables, proposing that public medical institutions cancel the markup on medical consumables by the end of 2019 and gradually adopt a centralized procurement approach (21). As early as July 2018, the healthcare reform in Guangdong province proposed that all public hospitals in Guangdong province would cancel the markup on medical consumables by the end of 2018 (22). In October 2020, the Joint Procurement Office for High-Value Medical Consumables, established by the State Council, implemented a nationwide centralized quantity procurement of coronary stents (23). The price of stents has decreased from an average of around 13,000 yuan to just over 700 yuan, a decrease of about 93% (24), and the cost of coronary stents costs was fully covered by medical insurance (25).

To what extent have the zero-markup consumable policy and the national procurement policy reduced hospitalization costs? How did the public hospitals cope with the reduction in consumables revenue? There have been a lot of studies on the zero-markup policy and national procurement policy for drugs, but few have analyzed the impact of the zero-markup policy and national procurement policy for high-value consumables on hospitalization costs. This study used the expense data collected from inpatients undergoing coronary stenting in a tertiary hospital in Guangzhou, Guangdong Province, from 2018 to 2021, to evaluate the impact of the two reforms on the amount and structure of hospitalization expense.

## 3 Methods

### 3.1 Data

China’s reform to reduce the expense of medical consumables has gone through two stages. The first phase is to eliminate the markup on consumables prices (zero-markup policy). The second stage is to implement centralized procurement of high-value medical consumables (national centralized procurement policy) to reduce the price of consumables. Since the end of 2018, Guangdong Province in China has implemented a zero-markup policy and gradually eliminated the markup on the prices of medical consumables. At the beginning of 2021, the national centralized procurement policy was implemented nationwide, and low-priced consumables procured through centralized procurement were introduced into public hospitals. The data for this study was sourced from the case management system of Guangdong Second Provincial General Hospital (GSPGH), a tertiary public hospital. The study included all 3,134 patients who underwent coronary stenting procedures at the hospital over a 48-month period, from January 2018 to December 2021. All patients had a discharge diagnosis of ischemic heart disease (ICD-10 codes: I20–I25). Using the GSPGH case management system, we collected detailed expense information, along with demographic and sociological characteristics for each patient, to support our analysis. After the preliminary data check, no null data was found in the dataset. We selected the healthcare category from the Consumer Price Index (CPI) for residents of Guangzhou. Using the 2018 CPI as the baseline, we adjusted the data for 2019, 2020, and 2021 accordingly.

### 3.2 Variables

The primary outcome variables of this study focus on the changes in total hospitalization expense, the structure of total expense, and the proportion of each structure in the total expense after the policies were implemented. The total hospitalization expense is divided into 6 categories, including western medicine expenses, diagnostic expenses (including pathology diagnostic expenses, laboratory diagnostic expenses, imaging diagnostic expenses, and clinical diagnostic project expenses), treatment expenses (including general treatment operation expenses, non-surgery treatment project expenses, and general medical service expenses), surgery expenses, medical consumables (including expenses for disposable medical consumables for examination and treatment), and other expenses (including traditional Chinese medicine expenses, nursing expenses, other expenses, rehabilitation expenses, and blood expenses). The proportion of each category in the total expense structure is calculated by dividing the sum of the expense of each category in the same month by the total expenses of all patients. The values of the total expense and each category in the total expense can directly reflect the quantitative changes in patient expenses before and after the reform. The proportion of each category in the total expense can reflect the structural changes in the patient inpatient expenses before and after the reform.

### 3.3 Study design and models

The study aims to describe the effect of three phases of coronary stent price reform. After normality tests, both the total expenses and the structure of total expenses are found to have a skewed distribution. Therefore, the median and the interquartile range (IQR) are used for descriptive statistics and interrupted time series modeling. The changing trends of total expense and its components from January 2018 to December 2021 are displayed by line graphs. The changing trends of proportion of each category in the total expense from January 2018 to December 2021 are also displayed by line graphs.

An interrupted time series (ITS) design is employed to evaluate the impact of the coronary stent price policy on inpatient expenses for patients (26). The ITS design is a quasi-experimental research design used to assess the effect of population-level health interventions at a specific time. It has been applied in various fields such as public health. The segmented regression function is fitted using the method of least squares. The basic form of the model is as follows:


$$\begin{array}{l} Y_{t}=\beta_{0}+\beta_{1}*t+\beta_{2}*\mathrm{refor}m_{1}+\beta_{3}*\mathrm{posttim}e_{1}+\\ \beta_{4}*\mathrm{refor}m_{2}+\beta_{5}*\mathrm{posttim}e_{2}+e_{t\text{.}} \end{array}$$

In this model, “Y_t_” represents the median or proportion of various expenses for each month, “t” (ranging from 0 to 47) is a time variable, which represents the months, “reform_1_” is a dummy variable for the zero-markup consumable policy intervention (pre-intervention 0, post-intervention 1), “reform_1_” is a time indicator variable for the zero drug mark-up policy (pre-intervention 0, post-intervention 0–35), “reform_2_” is a dummy variable for the policy adaptation period (pre-intervention 0, post-intervention 1), and “posttime_2_” is a time indicator variable for the policy adaptation period (0 pre-intervention, 0–17 post-intervention). The intercept in the regression equation is represented by $\beta_{0}$, $\beta_{1}$ represents the natural trend of expenses over time before the intervention, $\beta_{2}$ represents the immediate effect of the implementation of the policy relative to $\beta_{0}$, $\beta_{3}$ represents the long-term effect of zero-markup consumable policy relative to $\beta_{1}$, $\beta_{4}$ represents the immediate effect of national procurement policy implementation, and $\beta_{5}$ represents the change in the slope of the expense over time after the implementation of national procurement policy.

This study used R software (version: 4.3.1) for descriptive statistics and time series analysis. We address robustness issues by testing the underlying assumptions of the model. Before fitting the piecewise regression, we assess the stationarity and seasonality of the time series by analyzing the truncation and tailing characteristics in the autocorrelation function (ACF) and partial autocorrelation function (PACF) plots (27). After fitting the piecewise regression model, we conduct tests for heteroscedasticity and autocorrelation of the model residuals. The Breusch–Pagan test is employed to evaluate the presence of heteroscedasticity; if the *p*-value of the Breusch–Pagan test is less than 0.05, it indicates the existence of heteroscedasticity. In cases where heteroscedasticity is detected in the model residuals, we utilize robust standard errors to address this issue. Furthermore, we employ the Durbin–Watson test to assess autocorrelation in the model residuals; a *p*-value less than 0.05 suggests the presence of autocorrelation (26). If autocorrelation is identified in the model residuals, we apply generalized least squares estimation to correct the model’s regression coefficients, specifically using the Prais–Winsten regression method for this adjustment (28).

## 4 Results

### 4.1 Descriptive analysis

#### 4.1.1 Median of expenses

**Figure 1:**
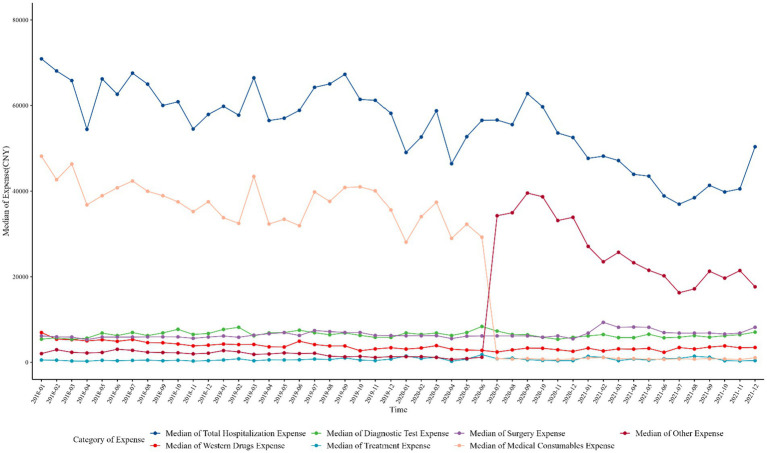
The time trend of the median of expenditures.

After the implementation of the zero-markup consumable policy, the total hospitalization expense, western drugs expense, medical consumables expense, and other expenses decreased, while the diagnostic test expense, treatment expense, and surgery expense increased. After the implementation of the national procurement policy for coronary stents, the trends of total hospitalization expense, western drugs expense, and medical consumables expense continued to decline, with the most significant changes in the expense of medical consumables. Meanwhile, the expense of treatment and surgery continued to show an upward trend. However, the expense of diagnostic tests has decreased; and of others has increased rapidly, which showed a complementary relationship with the decrease in the expense of medical consumables, as shown in Table 1 and Figure 1.

**Table 1:** Median and interquartile range of expenditures before and after reform.

Structure of expense, Median (Q1, Q3), CNY	Jan 2018 to Dec 2018 (*before reform*)	Jan 2019 to Jun 2020 (*reform_1_*)	Jul 2020 to Dec 2021 (*reform_2_*)
	*N* = 753	*N* = 1,192	*N* = 1,189
Total hospitalization expense	63124.98 (50029.33, 86043.21)	58332.50 (44685.75, 79798.57)	48266.34 (36941.31, 65924.87)
Western drugs expense	5007.86 (3287.64, 8071.78)	3588.26 (2389.07, 5942.01)	3171.85 (1993.31, 4887.27)
Diagnostic test expense	6300.29 (4778.40, 8626.44)	6779.84 (4883.52, 9218.17)	6202.79 (4783.87, 8002.44)
Treatment expense	416.52 (151.24, 1032.78)	654.57 (208.84, 2055.34)	682.47 (201.02, 2320.86)
Surgery expense	5912.50 (5262.50, 8363.00)	6288.54 (5570.81, 8770.47)	6830.51 (5492.48, 8789.89)
Medical consumables expense	39119.52 (31298.06, 56217.92)	34205.32 (25673.13, 52210.94)	785.79 (485.89, 1287.21)
Other expense	2427.11 (1435.66, 3967.22)	1518.61 (876.76, 2791.10)	28350.39 (18130.75, 39876.91)

In 2018, 1 USD ≈ 6.6174 CNY.

#### 4.1.2 Proportion of expenses

**Figure 2:**
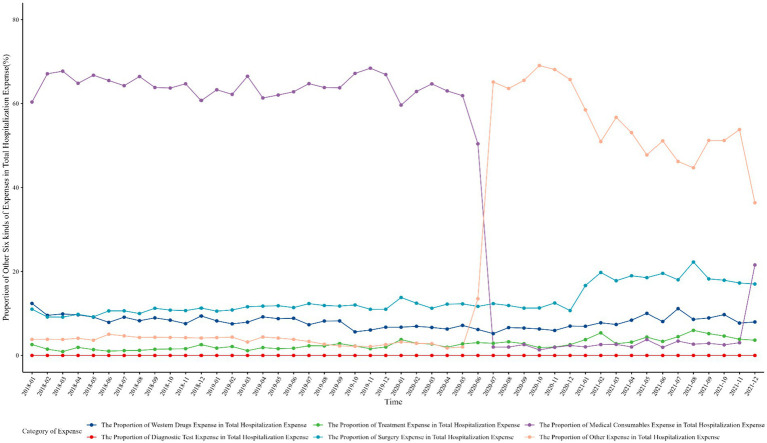
The time trend of the proportion of other six kinds of expenses in total hospitalization expense.

After the implementation of the zero-markup consumable policy, the proportions of diagnostic test expense, treatment expense, and surgery expense in total hospitalization expense increased and showed a consistent increase trend with the median. Meanwhile, the proportions of western drugs expense, medical consumables expense, and other expenses in total hospitalization expense decreased. After the implementation of the national procurement policy for coronary stents, the proportion of western drugs expense remained stable and showed little change compared to the zero-markup consumable policy. The proportion of diagnostic test expense, treatment expense, and surgery expense continued to increase, but the increase in the proportion of diagnostic test expense was different from the median decline in diagnostic test expense, which may be related to the reduction in total hospitalization expense. The proportions of medical consumables expense and other expense changed greatly, presenting a complementary relation, as shown in Table 2 and Figure 2.

**Table 2:** Proportion of other six kinds of expenses in total hospitalization expense before and after reform.

Structure of expense, Median (Q1, Q3), %	Jan 2018 to Dec 2018 (*before reform*) *N* = 753	Jan 2019 to Jun 2020 (*reform_1_*) *N* = 1,192	Jul 2020 to Dec 2021 (*reform_2_*) *N* = 1,189
Western drugs expense	7.73% (5.37%; 11.26%)	6.26% (4.39%; 9.13%)	6.52% (4.52%; 9.47%)
Diagnostic test expense	9.91% (7.28%; 12.91%)	11.39% (7.96%; 15.10%)	13.27% (9.64%; 17.52%)
Treatment expense	0.64% (0.27%; 1.44%)	0.99% (0.38%; 3.08%)	1.24% (0.44%; 4.65%)
Surgery expense	10.45% (8.37%; 12.56%)	12.17% (9.87%; 14.59%)	15.49% (11.55%; 20.21%)
Medical consumables expense	65.38% (57.34%; 72.09%)	63.13% (54.96%; 70.39%)	1.67% (1.03%; 2.68%)
Other expense	3.61% (2.23%; 5.60%)	2.46% (1.57%; 4.09%)	58.33% (46.05%; 67.93%)

According to the public notice of the national procurement bidding, the coronary stents centralized procured could not reach hospitals until early 2021. However, we found that there was a considerable change in the expense structure in July 2020 during the process of descriptive statistics. Therefore, we adjusted the intervention point of the policy to July 2020 and discussed the reason for this drastic change in detail in the discussion section.

### 4.2 Interrupted time series

#### 4.2.1 Effect on total expense and its structure

**Figure 3:**
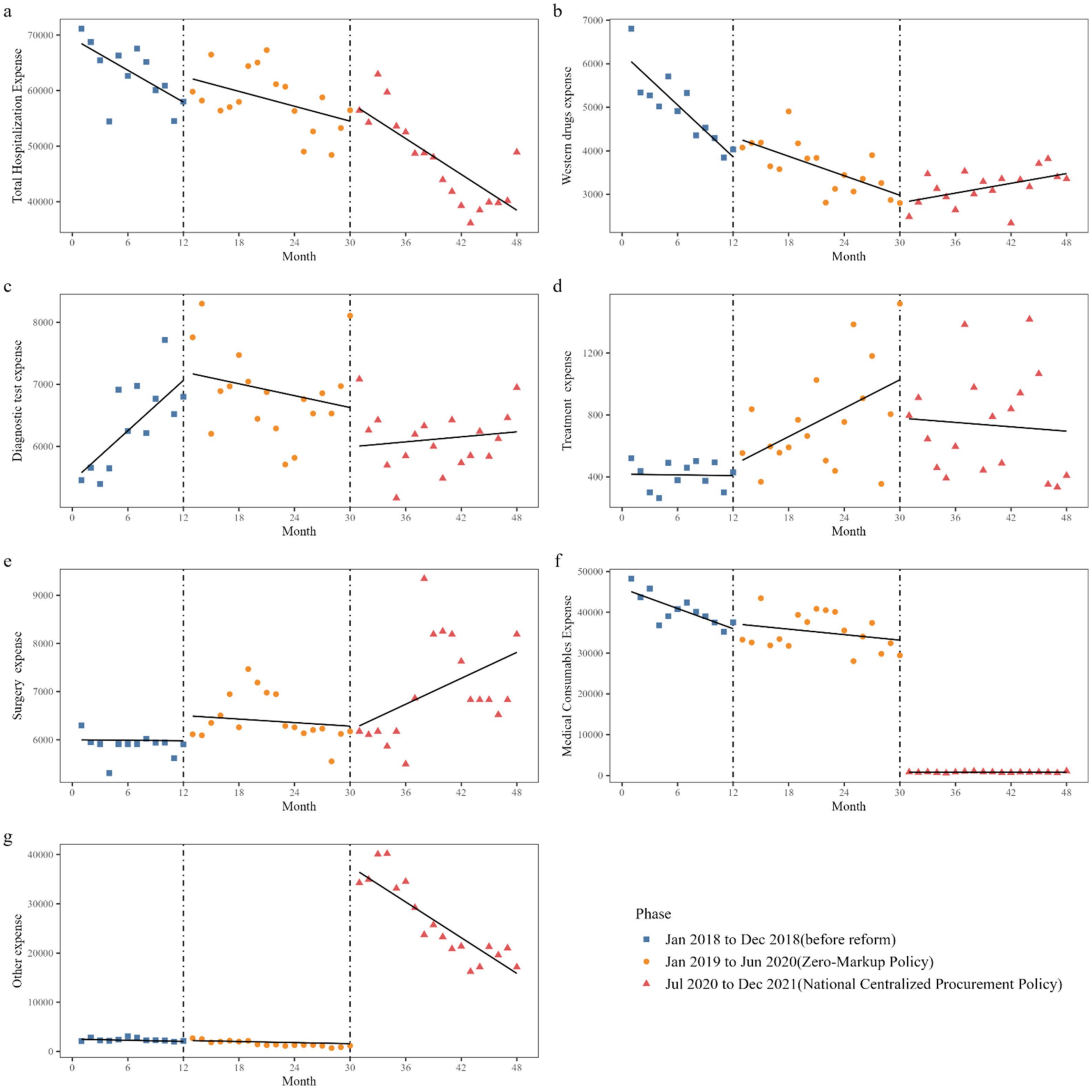
Trend in median of total expense and its structure of inpatients in GSPGH from 2018 to 2021. Median of expense of inpatient **(A)** total hospitalization expense, **(B)** western drugs expense, **(C)** diagnostic test expense, **(D)** treatment expense, **(E)** surgery expense, **(F)** medical consumables expense, **(G)** other expense.

The effects of the two reforms on total hospitalization expense and the various components of the total hospitalization expense are shown in Table 3 and Figure 3. At the initial level before the reform, the median of total hospitalization expense, western drugs expense, diagnostic test expense, treatment expense, surgery expense, medical consumables expense, and other expenses for patients were 69409.36-yuan, 6247.37-yuan, 5383.28-yuan, 417.51-yuan, 6000.92-yuan, 45872.03 yuan, and 2523.06 yuan, respectively.

**Table 3:** Effect on total expenditures and its structure.

Items of expense	Total hospitalization expense		Western drugs expense		Diagnostic test expense		Treatment expense		Surgery expense		Medical consumables expense		Other expense	
Method Employed	GLS		OLS		GLS		OLS + Robust SE		GLS + Robust SE		OLS + Robust SE		GLS + Robust SE	
	Coefficient	*p*-value	Coefficient	*p*-value	Coefficient	*p*-value	Coefficient	*p*-value	Coefficient	*p*-value	Coefficient	*p*-value	Coefficient	*p*-value
Intercept	69409.36	**<0.001**	6247.37	**<0.001**	5443.09	**<0.001**	417.51	**<0.001**	6000.92	**<0.001**	45872.03	**<0.001**	2523.06	**<0.001**
Slope pre-reform (β1)	−957.25	0.059	−198.97	**<0.001**	135.10	**0.026**	−0.74	0.923	−1.66	0.983	−824.93	**<0.001**	−39.76	0.358
Post-reform 1														
Change in level due to reform1 (β2)	5129.20	0.237	586.44	0.063	−30.26	0.953	99.70	0.380	512.42	0.407	1839.76	0.439	206.24	0.618
Change in slope due to reform1 (β3)	510.50	0.393	124.47	**0.002**	−166.98	**0.020**	31.29	0.073	−10.55	0.917	600.81	**0.037**	3.30	0.969
Post-reform 2														
Change in level due to reform2 (β4)	2692.03	0.490	−63.15	0.815	−587.62	0.213	−282.06	0.232	17.75	0.975	−32160.81	**<0.001**	34866.88	**<0.001**
Change in slope due to reform2 (β5)	−629.20	0.134	111.88	**<0.001**	45.33	0.349	−35.27	0.102	102.01	0.169	225.52	0.249	−1173.08	**<0.001**
*R*-squared	0.707		0.829		0.247		0.305		0.400		0.976		0.930	

In 2018, 1 USD ≈ 6.6174 CNY. Bold values indicate that the *p*-value is statistically significant at the 95% confidence level.

Before the start of reform 1, the median of total hospitalization expense, western drugs expense, and medical consumables expense decreased by 957.25 yuan (*p* = 0.059), 198.97 yuan (*p* < 0.001), and 824.93 yuan (*p* < 0.001) per month, respectively, while the median of diagnostic test expense increased by 150.35 yuan (*p* = 0.026) per month. There was no statistically significant change in treatment, surgery, and other expense per month.

At the initial level of reform 1, there was no statistically significant change in the level of all expenses including total hospitalization expenses. The decreasing trend in the median of medical consumables expense and western drugs expense decreased by 600.81 yuan (*p* = 0.037), 124.47 yuan (*p* = 0.002) per month, respectively, while the increasing trend in the median of diagnostic test expense decreased by 166.98 yuan (*p* = 0.002) per month. There was no statistically significant change in the trends of total hospitalization expenses, treatment, surgery, and other expense.

At the initial level of reform 2, the median of medical consumables expenses decreased by 32,160.81 yuan (*p* < 0.001), while the median of other expenses increased by 34,866.88 yuan (*p* < 0.001). There was no statistically significant change in the initial level of total hospitalization expense, western drugs expense, diagnostic test expense, treatment expense, or surgery expense. The decreasing trend in the median of western drugs expense decreased by 111.88 yuan (*p* < 0.001) per month due to reform 2. The sudden surge in the median of other expenses decreased by 1,173.08 yuan (*p* < 0.001) per month due to the reform 2. There was no statistically significant change in the trends of total hospitalization expense, diagnostic test expense, treatment expense, surgery expense, or medical consumables expense.

#### 4.2.2 Effect on proportion of total expense’s composition

**Figure 4:**
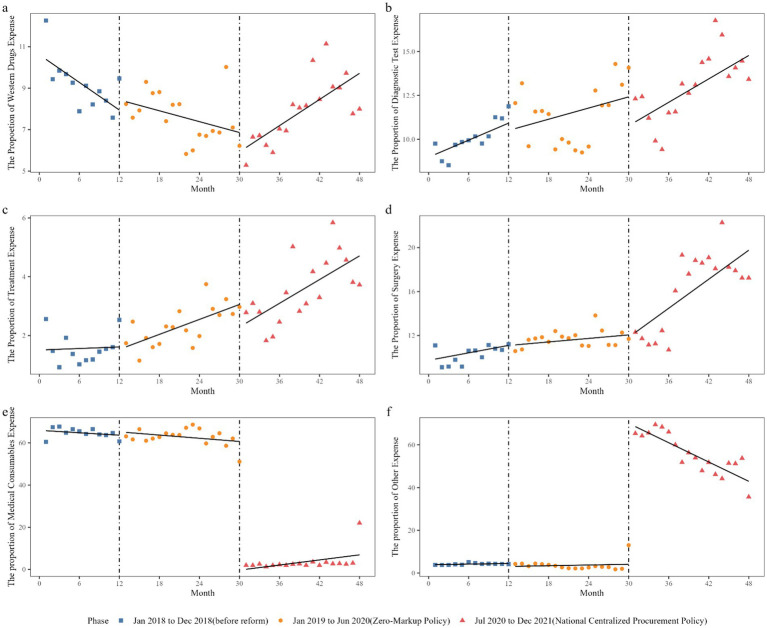
Trend in proportion of total expense’s compositions of inpatients in GSPGH from 2018 to 2021. Proportion of inpatient **(A)** western drugs expense, **(B)** diagnostic test expense, **(C)** treatment expense, **(D)** surgery expense, **(E)** medical consumables expense, **(F)** other expense.

The impact of the two reforms on the proportion of each component in the total hospitalization expense is shown in Table 4 and Figure 4. At the initial level before the reform, the proportion of patients’ expenses for western drugs, diagnostic tests, treatment, surgery, medical consumables, and other items to the total hospitalization expense was 10.61, 8.98, 1.51, 9.74, 65.97, and 3.88%, respectively. Before the reforms, the proportion of western drugs expense decreased by 0.22% per month (*p* = 0.017), and the trends of the proportion of other components did not show statistical significance.

**Table 4:** Effect on proportion of total expenditure’s composition.

Items of expense	Western drugs expense		Diagnostic test expense		Treatment expense		Surgery expense		Medical consumables expense		Other expense	
Method employed	OLS		GLS		OLS		GLS + Robust SE		OLS		OLS + Robust SE	
	Coefficient (%)	*p*-value	Coefficient (%)	*p-*value	Coefficient (%)	*p*-value	Coefficient (%)	*p*-value	Coefficient (%)	*p*-value	Coefficient (%)	*p*-value
Intercept	10.61	**<0.001**	8.98	**<0.001**	1.51	**<0.001**	9.74	**<0.001**	65.97	**<0.001**	3.88	**<0.001**
Slope pre-reform (β1)	−0.22	**0.017**	0.16	0.306	0.01	0.881	0.11	0.315	−0.19	0.535	0.04	0.012
Post-reform 1												
Change in level due to reform1 (β2)	0.62	0.451	−0.47	0.703	−0.003	0.996	−0.07	0.912	1.53	0.590	−1.37	0.191
Change in slope due to reform1 (β3)	0.13	0.194	−0.06	0.780	0.08	0.243	−0.06	0.648	−0.06	0.867	0.01	0.958
Post-reform 2												
Change in level due to reform2 (β4)	−0.63	0.381	−1.52	0.187	−0.72	0.120	0.17	0.854	−60.37	**<0.001**	64.38	**<0.001**
Change in slope due to reform2 (β5)	0.30	**<0.001**	0.11	0.422	0.05	0.260	0.39	**0.010**	0.66	**0.008**	−1.56	**<0.001**
*R*-squared	0.512		0.350		0.692		0.595		0.986		0.984	

Bold values indicate that the *p*-value is statistically significant at the 95% confidence level.

At the initial level of reform 1, the level and slope changes of the patients’ expenses for each component were not statistically significant. At the initial level of reform 2, there were significant changes in the proportion of patient expenses for medical consumables and other items, with a 60.37% decrease (*p* < 0.001) in the proportion of patients’ expenses for medical consumables and a 64.38% increase (*p* < 0.001) in the proportion of patients’ expense for other items. The slope change of the proportion of patient expense for western drugs was 0.30% (*p* < 0.001), for surgery was 0.39% (*p* = 0.018), for medical consumables was 0.66% (*p* = 0.008), and for other items was −1.56% (*p* < 0.001).

## 5 Discussion

The elimination of the medical consumables markup policy does not allow public hospitals to increase prices for inpatients to generate profits, while the national procurement policy greatly reduces the price of medical consumables purchased by public hospitals. These two reforms targeted the base and markup part of the consumable price paid by inpatients, respectively, and are expected to continuously reduce the expense of consumable and total hospitalization expenses for inpatients. However, our time series study found that the cost of consumables for inpatients with ischemic heart disease showed a significant downward trend before the reform. The cancellation of the consumable markup policy resulted in a median increase of RMB 1839.76 in consumables expense compared with the pre-reform period, with an average monthly increase of RMB 600.81, and the downward trend has slowed down. Further reforms, namely the national centralized procurement, immediately reduced the median consumable expense by 32,160.81 yuan, but afterward increased by an average of 225.52 yuan per month. Meanwhile, the proportion of consumable expenses did not significantly decrease before and after the cancellation of the consumable markup policy. However, after the implementation of the national procurement policy of coronary stents, the initial level dropped sharply by 60.37% and then increased by an average of 0.66% per month. The change in the median total hospitalization cost was similar to that of consumables, which also showed a downward trend before the reform, and then rose and fell after the reform, with a relatively small and insignificant change. The main reason is that after the reform, other costs increased significantly, making up for the gap caused by the reduction of consumables costs. Overall, the continuous reforms have significantly reduced the consumable cost, changed the cost structure, and alleviated the hospitalization cost of inpatients with ischemic heart disease.

After the cancellation of the consumable markup policy and the implementation of the national procurement of coronary stents, the average price of coronary stents decreased from approximately 13,000 yuan to around 700 yuan, a decrease of approximately 93%. However, does this price/expense reduction lead to a decrease in the quality of coronary stents? Coronary stent is high-value consumable and domestic stents were priced lower than imported stents before the implementation of these policies. Many patients believed that the quality of domestic stents was inferior to that of imported stents. With the improvement in living standards and increased health awareness among Chinese residents, patients are often inclined to use imported stents despite their high cost. Before the national procurement of coronary stents, the National Healthcare Security Administration conducted market research and determined that coronary stent technology had matured and there was little difference in stent quality between domestic and foreign suppliers. The State Drug Administration also issued a policy requiring local drug regulatory agencies to strengthen quality supervision of the selected stents that enter the market through national procurement (24, 29). Even before the implementation of the two policies for medical consumables, China had implemented national procurement for drugs and gained experience in quality regulation.

A single policy is not enough, only through the implementation of sustained reforms can the goal of reducing medical consumables costs be achieved. The median of total hospitalization expenditures has been consistently decreasing before and after reform. Although the interrupted time-series model did not attribute this downward trend to the reforms, the reduction in total hospitalization expenditures is beneficial to patients. Medical consumables expenditures varied greatly after the reforms. After Reform 1, the medical consumables expenditures continued the pre-reform downward trend. After Reform 2, the median consumables cost trend was relatively stable. The changes in consumables costs between the two reforms were quite different. The zero-markup policy mainly changed the trend of consumables costs, resulting in a long-term effect. The consumables centralized procurement policy changed the level of medical consumables expenditures, resulting in an instant effect. We believe that the cancellation of the consumables’ markup is a phased process. The medical consumables expenditures were decreasing before reform 2, but the trend decreased after the zero-markup policy was implemented. This does not seem to be in line with the reform, but we believe that hospitals have made changes earlier than policy changes and have started to adapt to possible policy changes. After the implementation of the centralized procurement policy for stents, the median of medical consumables expenditures of patients decreased significantly, but the long-term trend of medical consumables expenditures was not statistically significant. Therefore, the impact of centralized procurement policy on medical consumables expenditures was instantaneous. The national centralized procurement achieved the lowest consumables cost for patients all at once.

How did the hospital deal with a significant reduction in coronary stent revenue? In terms of income target theory, both the government and hospitals are expected to find ways to compensate for the decreased income. According to the clear provisions of the consumable reform, medical service expenditures are expected to be increased to subsidize part of the hospital’s income loss caused by the cancellation of the consumable’s markup. Therefore, as expected, patient surgery and treatment expenditures should have increased. The median of patients’ treatment expenditures and surgery expenditures both showed an upward trend, but according to interrupted time series analysis, the changes in patients’ treatment and surgery expenditures cannot be attributed to the reform. From the descriptive statistics, it can be seen that the increase in treatment expenditures is only a few tens of yuan, and the increase in surgery expenditures is only a few hundred yuan. Since the implementation of the comprehensive reform of medical consumables, the median and proportion of western drugs and diagnostic test expenditures in total expenses have shown a stable trend. The previous drug comprehensive reform has sufficiently suppressed the redundancy in the previous patient drug expenditures. Diagnostic test expenditures have also remained stable without significant fluctuations. In summary, the hospital’s increase in revenue through adjusting medical service prices was far from sufficient to compensate for the revenue losses caused by the reform of medical consumables. In previous studies on the zero markup policy for pharmaceuticals, it was similarly noted that the cancellation of the markup policy results in an increase in healthcare service expense or other related expenses (30, 31). One study indicated that after the implementation of the zero markup policy for pharmaceuticals, the expenses of Western medicine for patients with chronic renal failure decreased, while the costs of traditional Chinese medicine increased, as the markup on Chinese medicine still existed. This is similar to our findings, where the costs of consumables decreased while other expenses increased (32).

The public hospitals themselves have had to do their best to find ways to offset the loss of revenue. Contrary to our expectations, other expenditures should remain stable, but we found that before the reform and after the consumable’s markup was canceled, other expenditures remained stable. However, after the national procurement of coronary stents, other expenditures underwent a drastic change. The main reason for this is that in the past, expensive stents resulted in high expenditures. To keep the total expenditures within the range acceptable to patients and save patients’ medical expenditures, doctors would choose relatively simple surgery procedures. However, due to the significant reduction in coronary stents expense and the total hospitalization expenses being much lower than the maximum amount set by the health insurance fund, doctors can now choose more precise and detailed surgery methods, such as using intravascular ultrasound (IVUS), optical coherence tomography (OCT), fractional flow reserve (FFR), and other auxiliary tools to perform precise surgery treatments. These auxiliary measures can help the surgeon better evaluate the nature, severity, calcification, and diameter of the blood vessels, and choose appropriate stents; evaluate whether the stent is attached to the wall, whether the ends of the stent are symmetrical, and whether the lesion is completely covered; evaluate the situation of re-narrowing within the stent, clarify the cause, and guide treatment, making surgery treatment more scientifically based and effective. This may be an important factor in the increase in other expenditures while stent costs are decreasing.

Compared with other countries internationally, there are still some unreasonable aspects in the patient cost structure after the reform. A study in the United States showed that from 2010 to 2013, the average hospitalization expenditures for patients with ST-segment elevation myocardial infarction (STEMI) was $19,327, and the average hospitalization expenditures for non-ST-segment elevation myocardial infarction (NSTEMI) was $18,465, among 11,969 patients who received percutaneous coronary intervention from 233 hospitals. Of the total expenditures, 45% of the expenditures were related to catheterization laboratory expenses (including implanted devices), 22% to room and board, 22% related to room and board, 14% related to supplies, and 9% related to pharmacy (19). In a study conducted in nine countries and regions in Europe, including England, France, Germany, Hungary, Italy, Norway, Poland, Spain, and Denmark, and 45 hospitals on the hospitalization expenditures of patients with myocardial infarction, coronary stenting was performed in the sample hospitals in Norway, Italy, and France. Among them, the patients’ expenditures in France accounted for 7.55% of the hospitalization expenditures for diagnosis, and 46.45% for daily treatment or interventional treatment, including 22.20% for medical consumables, 22.78% for drugs, and 23.22% for hospital management. The patients’ expenditures in Italy accounted for 4.25% of the hospitalization expenditures for diagnosis, 41.48% for daily treatment or interventional treatment, including 30.70% for medical consumables, 9.35% for drugs, and 44.92% for hospital management. The patients’ expenditures in Norway accounted for 6.25% of the hospitalization costs for diagnosis, and 74.66% for daily treatment or interventional treatment, including 27.38% for medical consumables, 7.58% for drugs, and 11.51% for hospital management (33). A study in Malaysia found that regardless of whether it was a teaching hospital or a general hospital, the item that accounted for the highest proportion of patients’ expenditures was still medical consumables. In teaching hospitals, the second largest proportion was maintenance and usage of patients’ expenditures for the cardiac catheterization laboratory, while in general hospitals, the second largest proportion was the salaries for doctors (34). In the aforementioned countries, American patients spent more on intervention in the medical process, European patients paid hospital overheads, and although Malaysian patients spent a lot on medical consumables, their proportion of medical consumables was lower than that of China, and medical service fees and maintenance usage fees were much higher than in China. In this study, we did not find related details, but we speculate that this may be related to the previously inflated expenditures of consumables and the subsequent skyrocketing of other expenditures after the reform.

Based on domestic and foreign experiences, we have the following policy suggestions for further consumable price reforms. First, continuously adjust medical service prices to compensate for the loss of revenue caused by consumable reforms. Compared to drug price reforms, the compensation policy for consumable price reforms is unclear. The government has not provided corresponding financial subsidies for consumable price reform, nor has it specified the proportion and method of medical insurance reimbursement. As a result, the revenue loss caused by consumable price reform is mainly borne by hospitals, forcing them to find ways to increase revenue from other sources. Therefore, it is necessary to learn from the experiences of drug price reform and implement the government’s commitment to increasing medical service income. Specifically, the government should either provide a 5% subsidy for the eliminated markup on consumables, or the medical insurance fund should adjust the price of surgical services to fully compensate for the revenue losses incurred by hospitals due to the elimination of the consumable markup and centralized procurement. Second, reform should not only reduce costs, but also focus on healthcare quality. Under the guidance of relevant national policies, the current hospital charging details are stricter, the management of medical consumables is more standardized, and details that were previously overlooked have now received attention. When considering medical expenditures, we cannot simply look at the absolute value but must consider the medical effects and treatment benefits from multiple dimensions and aspects. Therefore, we believe that the reform should provide a better policy environment for medical behavior, allowing doctors to use more precise auxiliary methods to improve surgery results. In other words, the health administrative authorities must closely monitor the quality of coronary stenting procedures, including indicators such as mortality rates, readmission rates, postoperative complication rates, and the satisfaction of both doctors and patients (35), in order to prevent a decline in medical quality.

This article has several limitations in the following aspects. First, a single-center study in a sample hospital may have a bias in terms of research results. Although a hospital’s study can provide a very detailed description of policy effects and response strategies, there are significant regional, urban–rural, and typological differences in China’s public hospitals, and the research results of a comprehensive hospital in the southeastern coastal region may not reflect the situation of specialized hospitals in underdeveloped areas of the mainland. Therefore, it is necessary to collaborate with more hospitals to conduct representative multicenter studies nationwide. Second, there is a lack of research on individual expense changes and their influencing factors. This study included 3,134 patients but analyzed the impact of policy changes on medical consumable expenses and total hospital expenses at the median level. In reality, individual medical consumable costs and total hospitalization costs vary greatly and were influenced by factors such as medical condition, insurance, and region. In the next step, we will analyze more detailed cost changes and influencing factors at the individual level.

## 6 Conclusion

Using an interrupted time series design, we evaluated the impact of the zero consumables markup policy and centralized coronary stent procurement on inpatients who underwent coronary stenting. The analysis of interrupted time series revealed that the elimination of the consumable markup policy had a limited effect on the level and trend of total hospitalization expenses. However, the implementation of the national procurement policy for coronary stents led to a significant reduction in medical consumables expenses. But for hospital, the loss of incoming that these policies make cannot be compensated by the increase of medical service fees. Future reforms should focus on adjusting medical service prices to compensate for the revenue losses due to these reforms.

## Data Availability

The original contributions presented in the study are included in the article/Supplementary material, further inquiries can be directed to the corresponding authors.
