# Supplementary material for: Impact of zero-markup consumable policy and national procurement of coronary stents on hospitalization expenses: an interrupted time series analysis
Source: Front Public Health. 2025 Jan 30;13:1364116. doi: 10.3389/fpubh.2025.1364116 (PMC11821580; doi:10.3389/fpubh.2025.1364116)
Supplement: Supplementary file 1 [file Data_Sheet_1.PDF]

Appendix: residual normality tests for all models

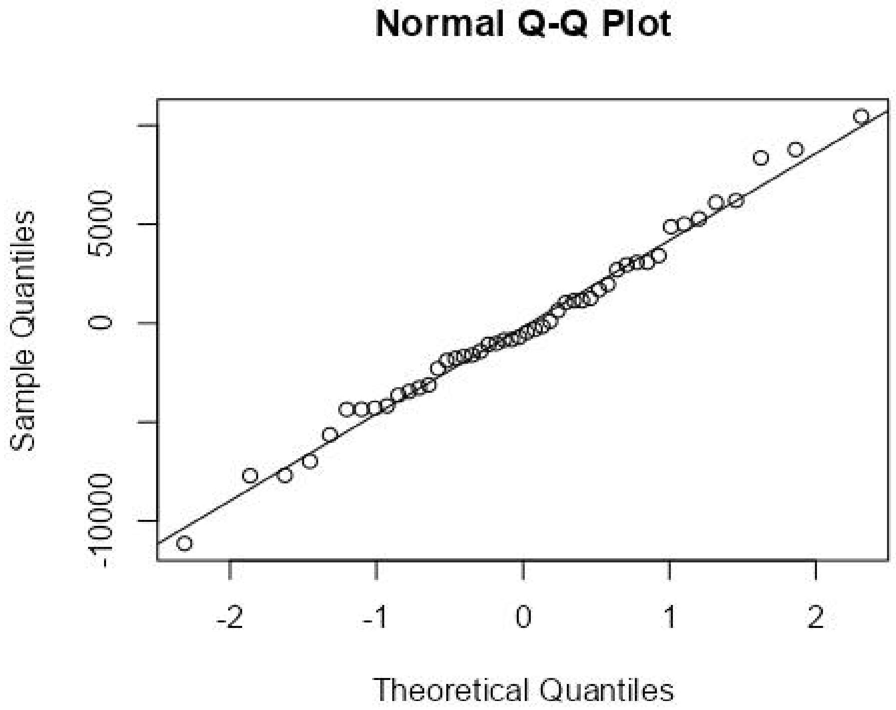

Fig.1 Q-Q plot of residuals of Total hospitalization expense Model.

It can be seen from Fig.1 that the residuals of the model show a normal distribution

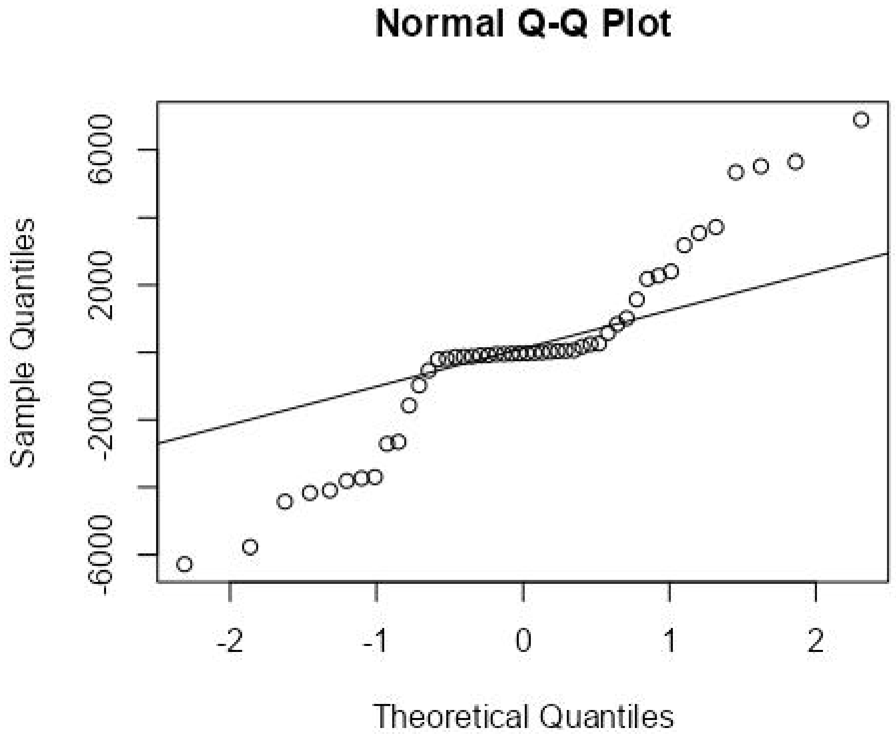

Fig.2 Q-Q plot of residuals of Medical consumables expense Model.

From Figure 2, it can be seen that although the residuals of the model do not conform

to normality, the problem can be solved to some extent by the use of robust standard errors in building the model.

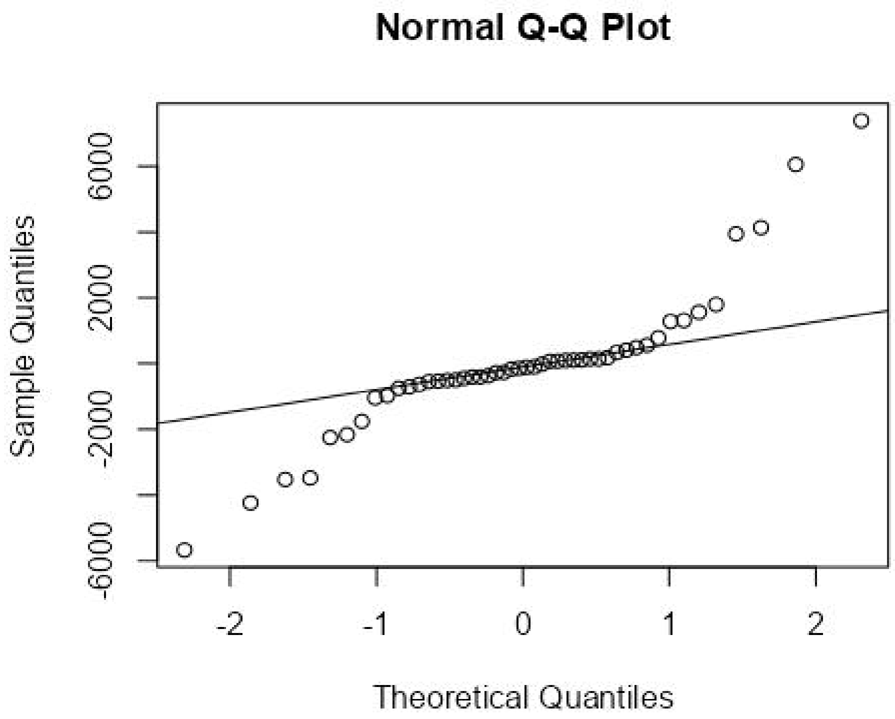

Fig.3 Q-Q plot of residuals of Other expense Model.

From Figure 3, it can be seen that although the residuals of the model do not conform to normality, the problem can be solved to some extent by the use of robust standard errors in building the model.

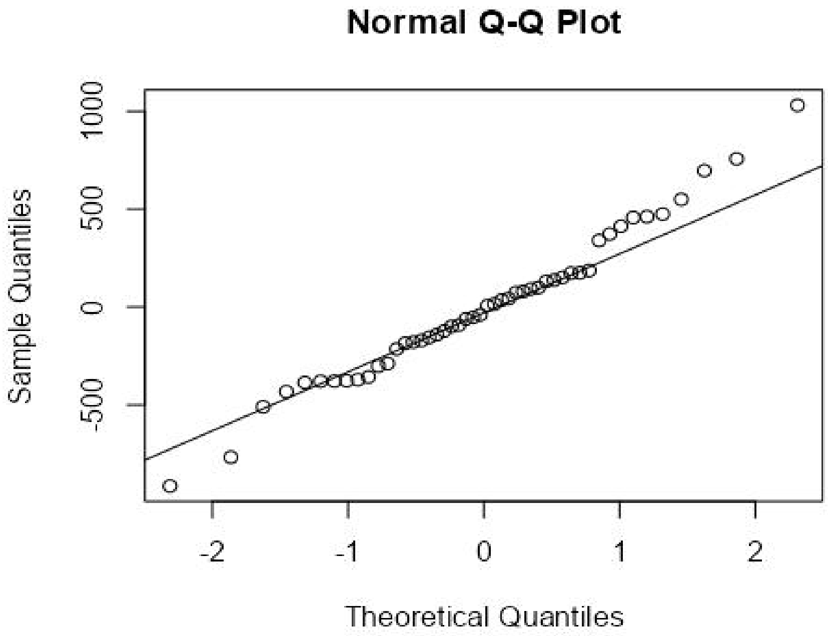

Fig.4 Q-Q plot of residuals of Western drugs expense Model.

It can be seen from Fig.4 that the residuals of the model roughly satisfy a normal

distribution

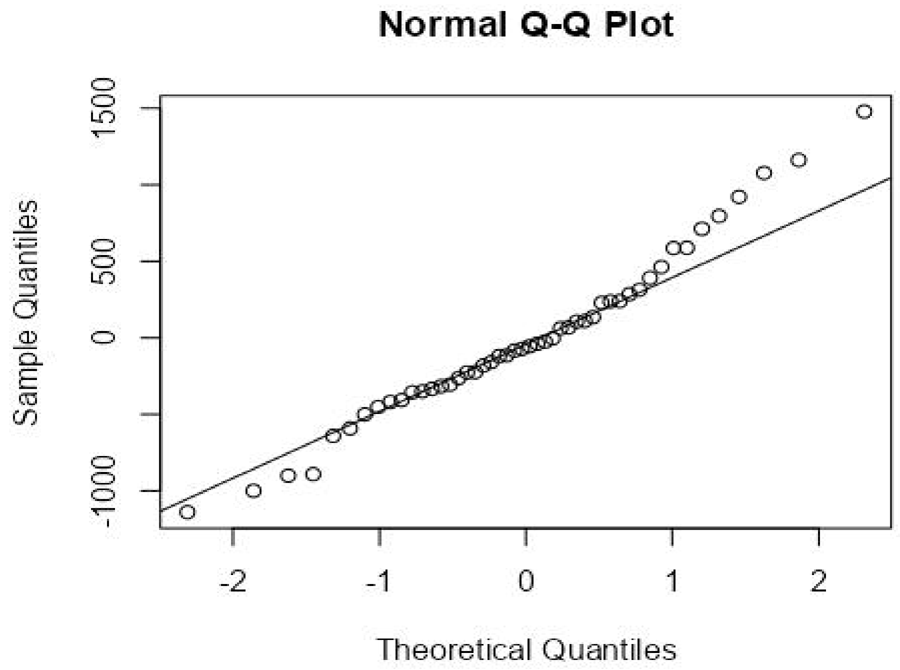

Fig.5 Q-Q plot of residuals of Diagnostic test expense Model

It can be seen from Fig.5 that the residuals of the model roughly satisfy a normal distribution

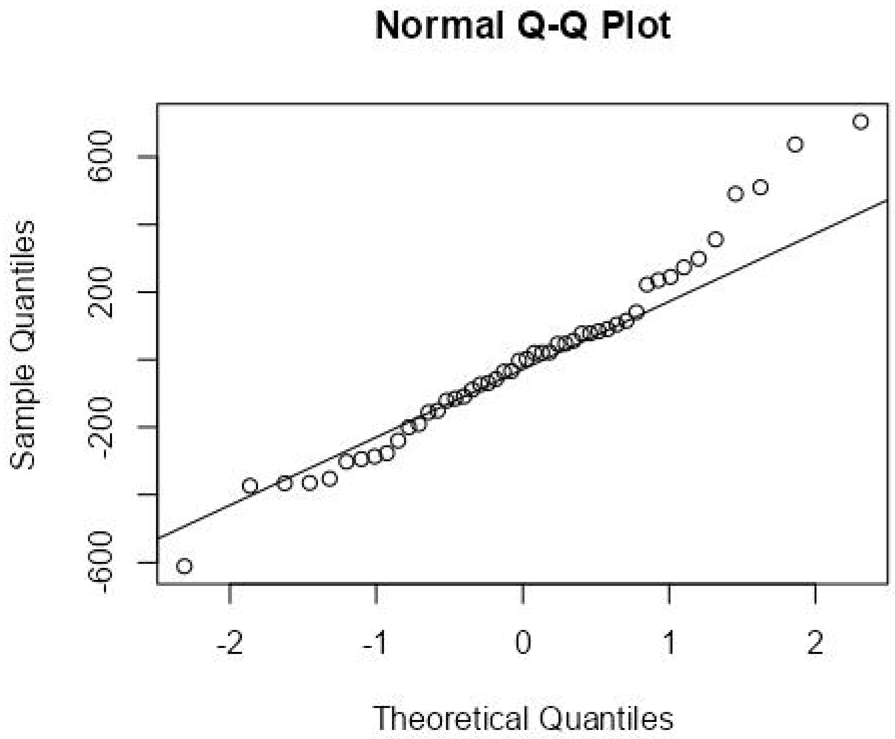

Fig.6 Q-Q plot of residuals of Treatment expense Model

It can be seen from Fig.6 that the residuals of the model roughly satisfy a normal

distribution

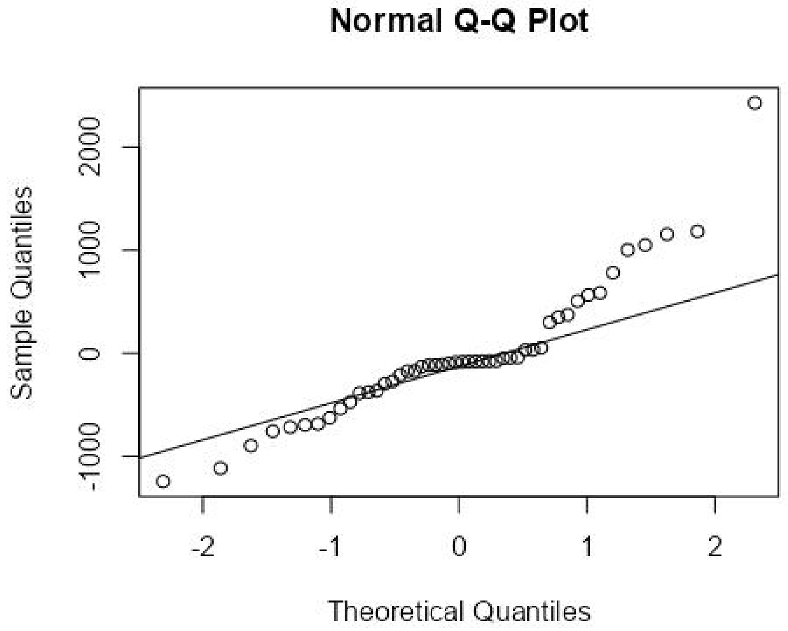

Fig.7 Q-Q plot of residuals of Surgery expense Model

From Figure 7, it can be seen that although the residuals of the model do not conform to normality, the problem can be solved to some extent by the use of robust standard errors in building the model.

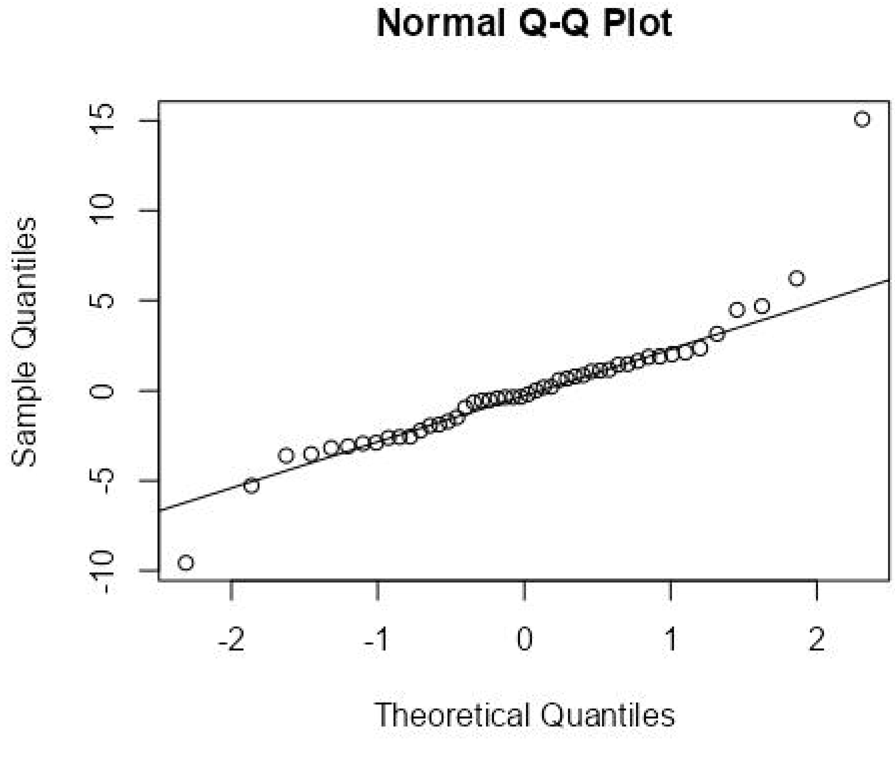

Fig.8 Q-Q plot of residuals of Proportion of medical consumables expense Model

It can be seen from Fig.8 that the residuals of the model roughly satisfy a normal

distribution

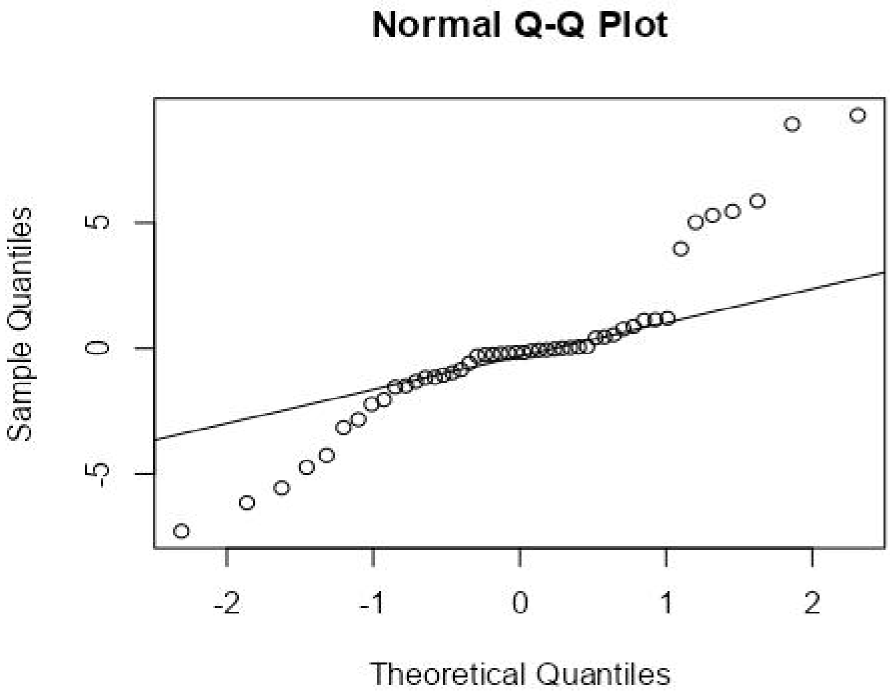

Fig.9 Q-Q plot of residuals of Proportion of other expense Model

From Figure 9, it can be seen that although the residuals of the model do not conform to normality, the problem can be solved to some extent by the use of robust standard errors in building the model.

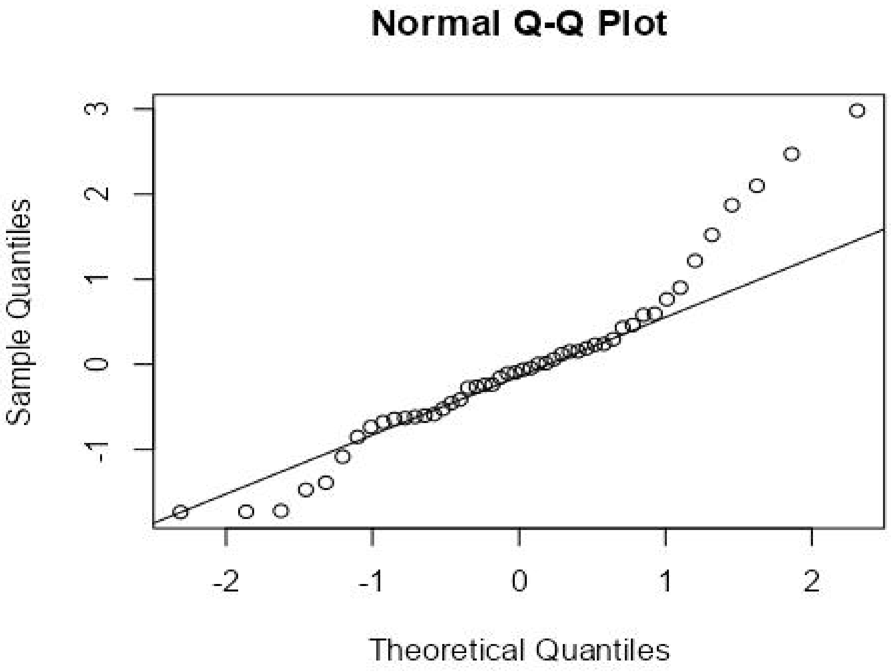

Fig.10 Q-Q plot of residuals of Proportion of western drugs expense Model

It can be seen from Fig.10 that the residuals of the model roughly satisfy a normal

distribution

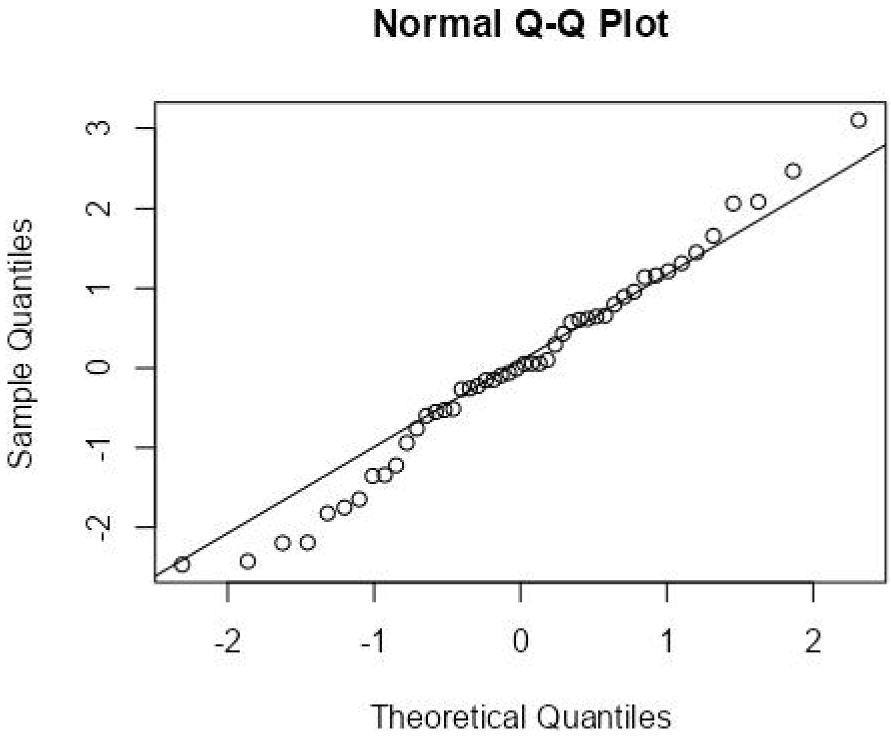

Fig.11 Q-Q plot of residuals of Proportion of diagnostic test expense Model

It can be seen from Fig.11 that the residuals of the model roughly satisfy a normal distribution

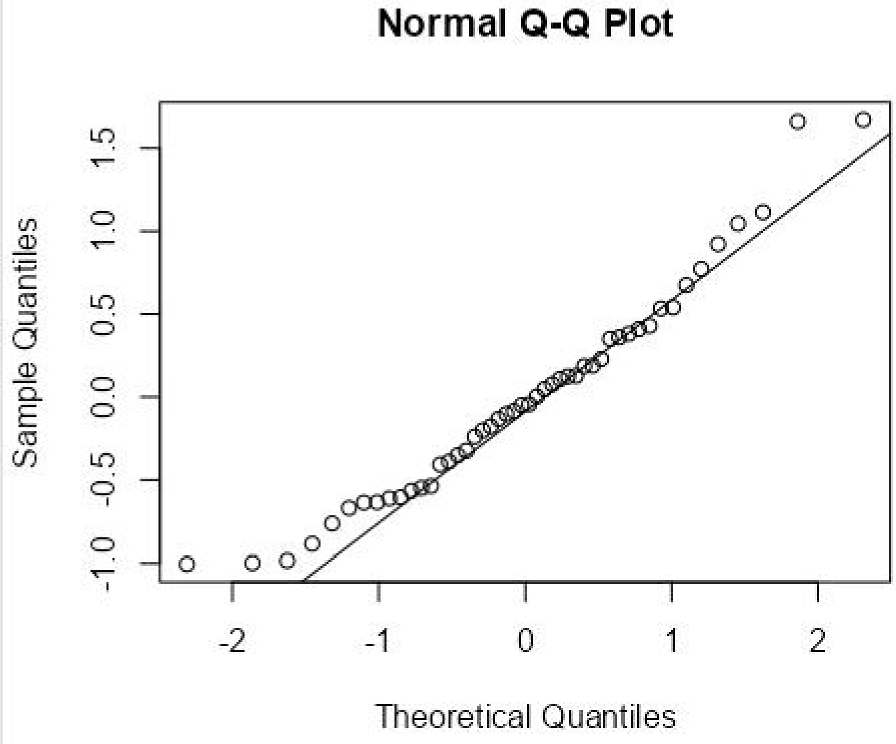

Fig.12 Q-Q plot of residuals of Proportion of treatment expense Model

It can be seen from Fig.12 that the residuals of the model roughly satisfy a normal distribution

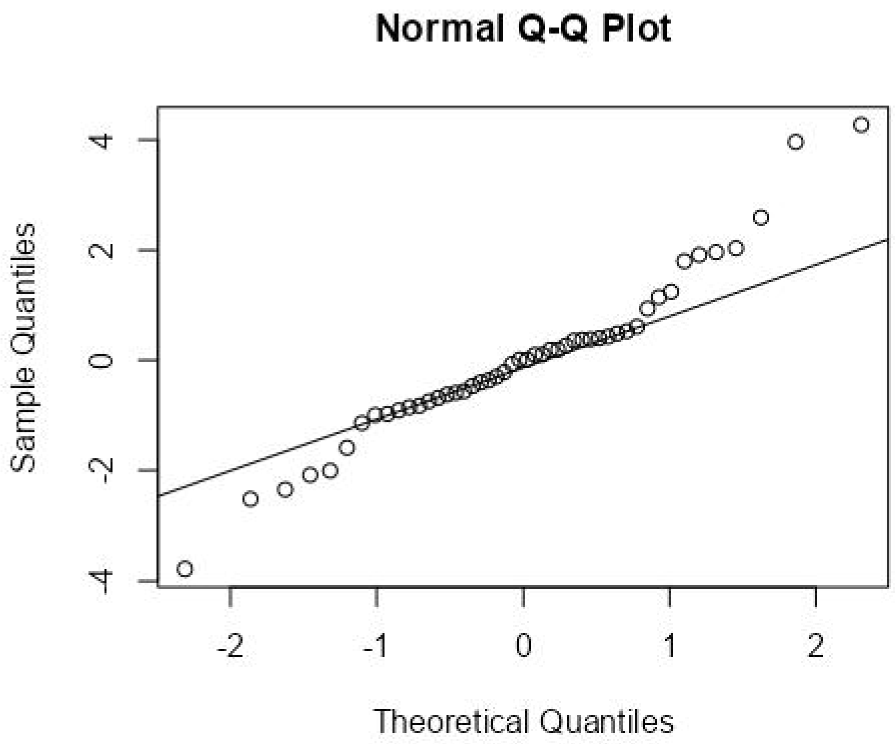

Fig.13 Q-Q plot of residuals of Proportion of surgery expense Model

It can be seen from Fig.13 that the residuals of the model roughly satisfy a normal distribution
